# ﻿*Derrislongiracemosa* (Fabaceae), a new species from Thailand with extraordinary limestone adaptations and the longest inflorescences ever recorded

**DOI:** 10.3897/phytokeys.261.156249

**Published:** 2025-08-05

**Authors:** Punvarit Boonprajan, Saruta Oncham, Yotsawate Sirichamorn

**Affiliations:** 1 Department of Biology, Faculty of Science, Silpakorn University, Sanam Chandra Palace Campus, Nakhon Pathom 73000, Thailand Silpakorn University Nakhon Pathom Thailand

**Keywords:** Endangered, IUCN, leaf anatomy, legume, Leguminosae, Millettieae, Papilionoideae, Thailand’s southwestern forest

## Abstract

A new species of *Derris* Lour. (Fabaceae), *Derrislongiracemosa* Boonprajan & Sirich., **sp. nov.**, is described as the species bearing the longest inflorescences recorded in the genus to date, reaching up to 155 cm in length. Endemic to limestone areas in southwestern Thailand, it is possibly the third known limestone-adapted *Derris* species. Compared with its closest morphological relative and a partially sympatric species, *D.solorioides*, *D.longiracemosa* exhibits a longer and differently structured inflorescence, lower numbers of ovules per ovary (fewer than five ovules), and flowers that turn pinker with maturity. Leaf anatomical study reveals several differences, such as the shape of epidermal cells, the presence of secretory-like cavities, an atypical stomatal type that occasionally occurs, the presence of schizogenous cavities in the midrib cortex, and the distinct absence of lysigenous cavities in the pulvini cortex, as well as a thicker mesophyll compared to *D.solorioides*. Molecular phylogenetic analysis using nuclear ITS and plastid *trnL-F* and *trnK-matK* sequences confirms all four sampled populations as a single, well-supported species, distinct from other *Derris* taxa but showing a close relationship with *D.rubrocalyx* only in Bayesian inference. This combination of morphological, anatomical, and molecular evidence supports the recognition of *Derrislongiracemosa* as a distinct species. A detailed description, distribution map, line drawing, photographs, and preliminary IUCN conservation status are provided.

## ﻿Introduction

Thailand harbors remarkable ecological diversity, with limestone habitats covering approximately 18% of the country, or around 93,000 km^2^ (Bolger and Ellis 2015). These habitats are globally recognized for their unique ecological characteristics, including high levels of plant endemism and many species at risk of extinction (Struebig et al. 2009). The rugged limestone outcrops provide a mosaic of microhabitats that support plant species specially adapted to nutrient-poor soils, sporadic water availability, and harsh climatic conditions (Clements et al. 2006; Batori et al. 2019; Kiew and Rahman 2021). In recent years, numerous new and often endemic taxa have been discovered in these ecosystems across several families, such as Balsaminaceae (Suksathan and Triboun 2009; Ruchisansakun et al. 2014), Begoniaceae (Phutthai and Sridith 2010; Phutthai and Hughes 2017), Gesneriaceae (Middleton and Möller 2012; Middleton and Triboun 2012), and Fabaceae (Burtt and Chermsirivathana 1971; Mattapha et al. 2017, 2023).

*Derris* Lour. is a genus within the family Fabaceae, tribe Millettieae. The “Flora of Thailand” (Sirichamorn 2020) officially recognizes seventeen species in the genus, with one more, *D.rubricosta* Boonprajan & Sirich., reported subsequently (Boonprajan et al. 2024). *Derris* species typically favor high levels of sunlight and thrive in semi-aquatic environments, often growing close to watercourses. Among these species, *D.tonkinensis* Gagnep., found in southern China, northern Vietnam, and northern Thailand, is unusual within the genus for its association with limestone habitats. This species lacks records from non-karst environments, and all known collections to date originate exclusively from limestone outcrops, suggesting ecological specialization to calcareous substrates. In 2014, *D.solorioides* Sirich. & Adema, a second limestone-dwelling species, was discovered in north-central Thailand (Sirichamorn et al. 2014). Subsequently, this species has also been documented in various limestone areas in Kanchanaburi Province, southwestern Thailand. Although a few populations (estimated at less than 2%) have been reported from non-limestone substrates, the vast majority are confined to karst landscapes, reinforcing its strong ecological association with limestone habitats. The observation of an additional limestone-associated taxon, which remained unidentified at the time of discovery, reinforces the view that such habitats promote lineage diversification in *Derris*. Edaphic specialization to karst substrates likely contributes to ecological segregation, setting the stage for divergence and eventual speciation within this complex landscape.

In Thailand’s rainy season (May to October) of 2020, during a field expedition in the limestone regions of Ratchaburi Province in southwest Thailand, the authors’ team discovered an unknown, non-flowering liana resembling *Derris* while traversing the trail to Phra Borommathat Bowonwisutthi Chedi at the summit of the limestone hill behind Wat (= temple in Thai) Khao Chong Phran, Photharam District, Ratchaburi Province. These plants initially appeared to be *Derrissolorioides* due to their similarity in terms of vegetative characteristics, habitat, and distribution. As a result, this incorrect data was included in the distribution of *D.solorioides* on page 412 of “Flora of Thailand”, vol. 4, part 3.2 (Leguminosae–Papilionoideae), published in 2024. Upon returning to the site in late February 2021, the team encountered a dried inflorescence bearing just three to five desiccated flowers and flower buds. This inflorescence displayed unique characteristics, including brachyblasts with a hairy rachis, lateral branches, pedicels, and calyces. It was clearly different from the typical paniculate inflorescence of *D.solorioides*, which lacks brachyblasts and boasts a comparatively more glabrous appearance in its inflorescence and floral components. In November and December of the same year, the researchers were able to collect more flowering specimens from this population. These specimens exhibited several notable characters in their reproductive structures, particularly in the longest inflorescence, reaching up to 155 cm, and the remarkable abundance of flowers on a brachyblast, with a maximum count of 16 flowers. Additional populations of this species were subsequently discovered in the vicinity, including Khao Pakarang, one of the larger limestone hills close to Ban Krang Substation within Kaeng Krachan National Park in Phetchaburi Province. Moreover, two more populations were found in non-limestone forests at Ban Krang Substation and in Ko Nok, Kaeng Krachan Dam rope bridge, near the tourist information center of Kaeng Krachan National Park, Phetchaburi Province. In the same period of the year, three non-flowering populations, vegetatively similar to both this unknown taxon and *D.solorioides*, were found in limestone hills of Ratchaburi and Phetchaburi provinces and also included in this study to determine their precise taxonomic status.

While the morphological traits of this species were recognized by the authors to be highly distinctive and thus potentially representing a novel scientific discovery, there remained uncertainties regarding its taxonomic position and a dearth of information on leaf anatomical features. This study aims to address these gaps by presenting a molecular phylogenetic analysis, as well as an examination of leaf anatomical and morphological characteristics. The full description, photographs, and line drawings of the new species, including a revised key to Thai species of *Derris*, are provided.

## ﻿Material and methods

### ﻿Sample collection, preparation, and morphological study

Four samples of the putative new *Derris* species (as *Derris* sp. in Table 1) were collected for comprehensive analysis, including morphology, anatomy, and molecular phylogeny. Voucher specimens were stored at the BKF herbarium, with duplicates sent to other herbaria such as K and L. For anatomical examination, mature leaflets per accession were preserved in 70% ethyl alcohol. Simultaneously, young leaves were collected and preserved in silica gel for DNA extraction. Voucher specimens were examined with a microscope. The species description followed the “Flora of Thailand” format (Sirichamorn 2020), which included detailed morphological measurements and comparative analysis based on *Derris* and *Derris*-like specimens from Thai herbaria (BK, BKF, PSU) and online images (K, L, P). To assess the status of three non-flowering specimens resembling *D.solorioides* (as Derriscf.solorioides in Table 1) in southwestern limestone habitats, young leaves of those specimens were also collected for DNA extraction. These samples were included in molecular phylogenetic analyses, comparing them with known sequences from the NCBI database of the type specimen of *D.solorioides* (Sirichamorn YSM2013-1), originally collected from a limestone area in Tham Phet–Tham Thong Forest Park in Nakhon Sawan Province, central Thailand.

**Table 1.** Species, localities, and vouchers of the material in Thailand used in the analyses.

Species	Locality	Code	Voucher specimen	Herbarium
*Derris* sp.	Wat Khao Chong Phran, Photharam District, Ratchaburi Province (Limestone area)	RP	YSM2021-36	BKF
*Derris* sp.	Khao Pakarang, vininity of Ban Krang substation, Kaeng Krachan National Park, Phetchaburi Province (Limestone area)	KP	YSM2023-1	BKF
*Derris* sp.	Ban Krang Camp, vicinity of Ban Krang Substation, Kaeng Krachan National Park, Phetchaburi Province (Non-limestone area)	PK	YSM2023-2	BKF
*Derris* sp.	Ko Nok, Kaeng Krachan Dam Rope Bridge, near tourist information center, Kaeng Krachan National Park, Phetchaburi Province (Non-limestone area)	PN	YSM2023-15	BKF
Derriscf.solorioides*	Wat Tham Mongkut (Khao Thalu), Chom Bueng District, Ratchaburi Province	RC	YSM2021-33	BKF
Derriscf.solorioides*	Khao Ngu Rock Park, Mueang Ratchaburi District, Ratchaburi Province	RM	YSM2021-34	BKF
Derriscf.solorioides*	Khao Nang Phanthurat Forest Park, Cha-am District, Phetchaburi Province	PC	YSM2021-35	BKF

Note: *the specimen had no flower at the time of collection.

### ﻿Assessment of conservation status

An initial evaluation of the species’ conservation status was conducted following the IUCN Categories and Criteria, in line with the latest guidelines from the IUCN Standards and Petitions Committee (2024). The extent of occurrence (EOO) and area of occupancy (AOO) were determined using GeoCat (Bachman et al. 2011).

### ﻿Molecular phylogenetic analyses

The selection of taxa for this investigation was based on the phylogeny outlined by Boonprajan et al. (2024). Additional DNA samples were obtained from dried young leaves of four populations of the putative new species and three populations of Derriscf.solorioides (Table 1) using the DNeasy Plant Mini Kit with a modified protocol (Qiagen, Hilden, Germany). We targeted two chloroplast regions, the *trnL-F* intergenic spacer (IGS) and *trnK-matK*, along with one nuclear marker, the internal transcribed spacer (ITS/5.8S), which have been widely used in legumes and shown to be informative in resolving species-level relationships within *Derris* and related genera. The PCR reaction mixture (25 µL) contained 1 µL (10 µM) of each forward and reverse primer, 12.5 µL GoTaq Green Master Mix (Promega), 2 µL (< 250 ng) of DNA template, and nuclease-free water. The PCR conditions followed a modified protocol by Hu et al. (2000) and Wojciechowski et al. (1993, 1999). Celemics, Inc. (http://www.celemics.com) purified and sequenced the samples. Each purified fragment underwent Barcode-Tagged Sequencing (BTSeq) with next-generation sequencing (NGS) technology for automated dsDNA sequencing. All newly generated sequences from this study have been deposited in GenBank and listed in Suppl. material 1.

Sequence alignments were performed using BioEdit v. 7.0.9 (Hall 1999) with CLUSTAL W multiple alignment (default settings; Thompson et al. 1994), followed by manual adjustment. Markers were initially aligned separately, and then a combined matrix was created by concatenating these datasets. Phylogenetic relationships were reconstructed through maximum parsimony (MP), maximum likelihood (ML), and Bayesian inference (BI). For MP analyses, trees were constructed using PAUP* v. 4.0a169 (Swofford 2002) with 10,000 replicates of random taxon additions, employing tree bisection–reconnection (TBR) branch swapping with the Multrees option activated. Bootstrap percentage analysis was calculated using the same settings (Felsenstein 1985) to assess MP clade support. The jModelTest v. 2 (Darriba et al. 2012) on the CIPRES web portal determined the best-fit substitution model based on Akaike Information Criterion (AIC) scores (Akaike 1974), with GTR+G selected. ML analyses were conducted using IQ-TREE v. 2.2.0 (Nguyen et al. 2015) with GTR+G partition models, and bootstrap clade support was calculated. Bayesian MCMC phylogenetic analyses used MrBayes v. 3.2.7a (Ronquist et al. 2012) on the CIPRES Science Gateway v. 3.3 (Miller et al. 2010) with a majority-rule consensus tree and 10,000,000 generations until stationarity, with MCMC sampled every 1,000 generations. BI clades were supported by posterior probabilities (PP).

### ﻿Leaf anatomical studies

All leaf samples used for anatomical investigation were collected during the same season across all populations to ensure comparability and consistency while minimizing potential seasonal variation. For each specimen shown in Table 1, three individuals per population were sampled, and from each individual, three leaflets from three mature leaves were fixed in 70% ethyl alcohol. Samples were prepared using a protocol modified from Johansen (1940) for paraffin embedding. Tissue dehydration was performed using the TBA series, followed by paraffin embedding. Transverse sections (15 µm thick) were prepared using a rotary microtome (Leica RM 2145) and double-stained with 1% Safranin O and 1% Fast Green, respectively.

Mesophyll from both the upper and lower surfaces of *Derris* sp. and Derriscf.solorioides was scraped using a razor blade, followed by epidermal bleaching with 10% sodium hypochlorite. After three distilled water washes, the samples underwent dehydration through a graded series of ethyl alcohol (with staining using 1% Fast Green after the 95% ethyl alcohol step) and a xylene series. The prepared samples were then mounted on slides using DePeX mounting media. All sectioned leaf parts and epidermal surfaces were digitally captured using an Olympus BX53 microscope equipped with a DP27 camera attachment. The anatomical features of each leaf were quantified using ImageJ (Rueden et al. 2017).

## ﻿Results

### ﻿Molecular phylogenetic analyses

**Figure 1.**
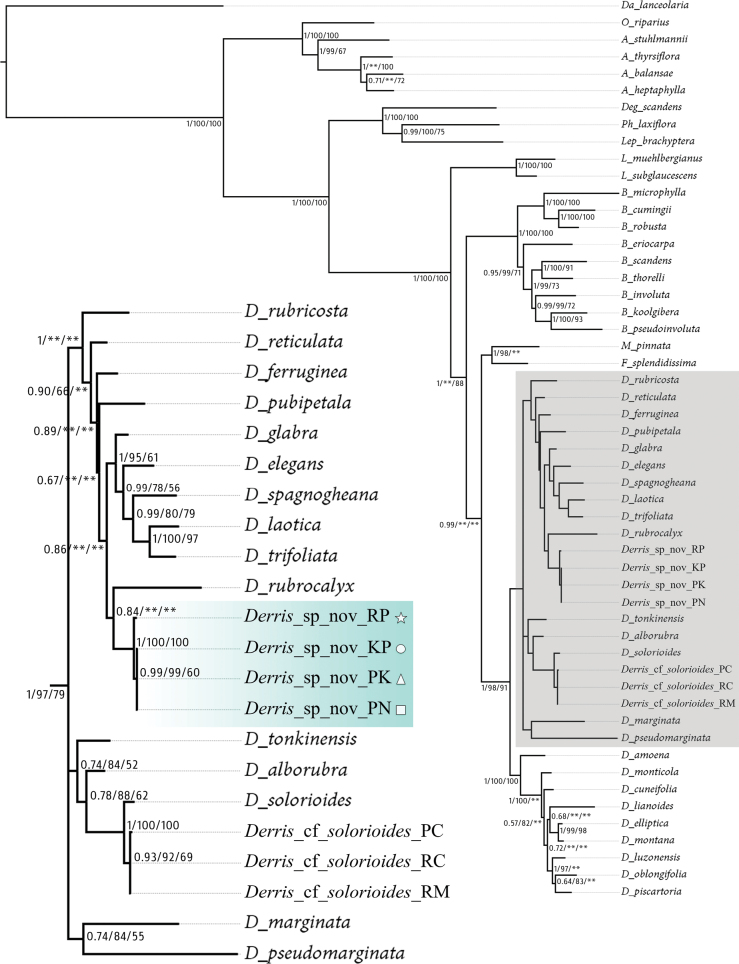
Bayesian consensus tree from Bayesian inference. The posterior probabilities (PP), bootstrap percentage support values of maximum likelihood (MLBS) and maximum parsimony (MPBS), respectively (** = MPBS and/or MLBS < 50%). Abbreviations and symbols after scientific names indicate locality codes according to Table 1 and Fig. 6.

Four samples of the putative new *Derris* species formed a monophyletic clade with strong support across all analyses (posterior probability = 1; bootstrap support for maximum likelihood = 100 and maximum parsimony = 100; Fig. 1). This result confirms that these samples belong to the same species. Additionally, they are resolved as sister to *D.rubrocalyx*, the N. Queensland and Papua New Guinean species, with relatively high support in the Bayesian analysis (posterior probability = 0.84), although this relationship is not strongly supported in ML and MP analyses. This incongruence may reflect phylogenetic uncertainty, possibly due to methodological differences or limited informative characters. Bayesian inference can detect weak signals more readily through its probabilistic framework, while ML and MP are more conservative and may be more affected by homoplasy. Nevertheless, the consistent recovery of this sister relationship, together with clear morphological distinctiveness, supports the recognition of the new species. In contrast, three non-flowering specimens formed a clade with *D.solorioides*, again with strong support across all analyses (posterior probability = 1; bootstrap support for maximum likelihood = 100 and maximum parsimony = 100; Fig. 1). This indicates that these three specimens also belong to *D.solorioides*.

### ﻿Leaf anatomical analysis

**Figure 2.**
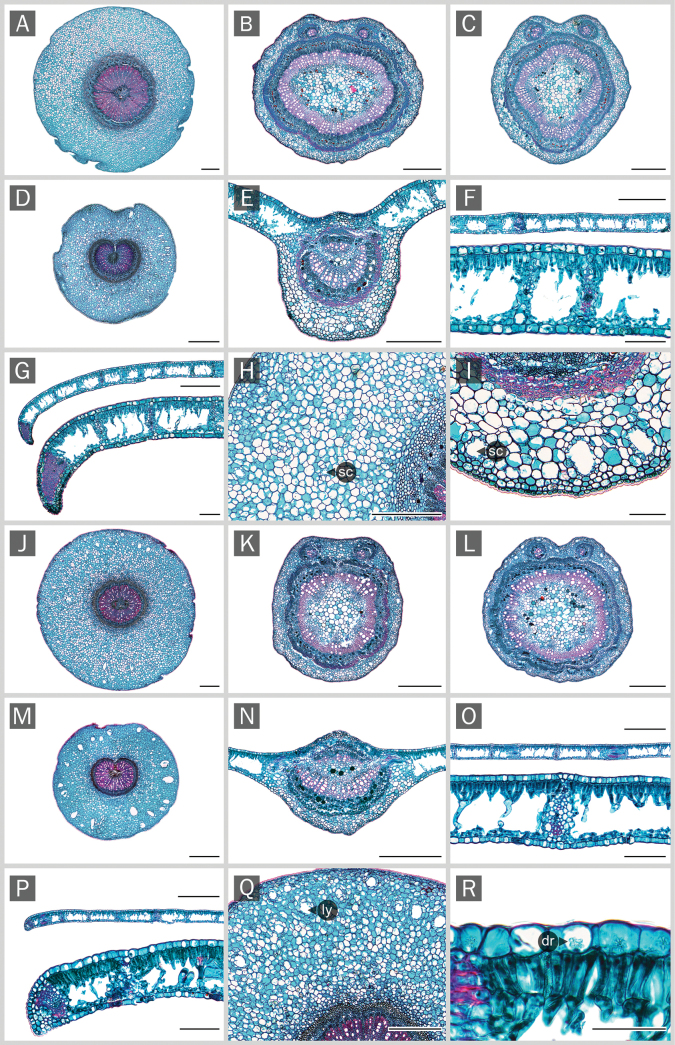
Comparative anatomical characters of leaf transverse sections. A, J, H, Q. Petiole pulvinus; B, K. Petiole; C, L. Rachis; D, M. Petiolule pulvinus; E, N, I. Midrib; F, O, R. Leaf blade; G, P. Leaf margin; A–I. The new *Derris* samples and J–R. *D.solorioides*. dr, druse crystal; sc, schizogenous cavity; ly, lysigenous cavity. Scale bars: 50 µm (R); 100 µm [G (below), F (below), I, P (below), O (below)]; 500 µm [(A–E, F (above), G (above), H, J–N, O (above), P (above), Q)].

**Figure 3.**
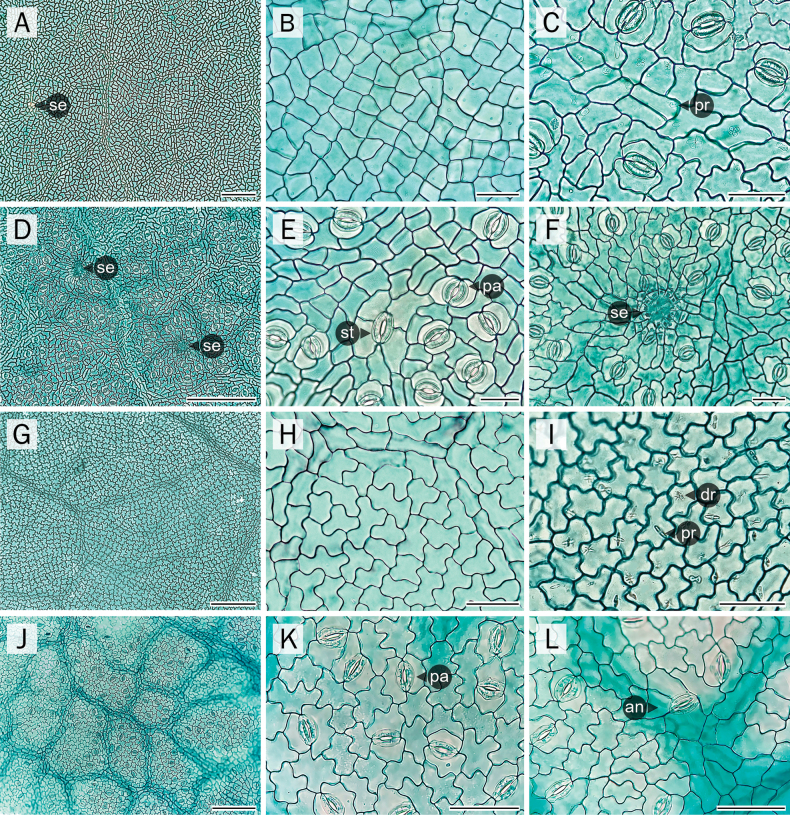
Comparative anatomical characters of leaf epidermis. A, B, G–I. Adaxial leaf epidermis; C–F, J–L. Abaxial leaf epidermis; A–F. The new *Derris* samples; G–L. *D.solorioides*. an, anomocytic stoma; dr, druse crystal; pa, paracytic stoma; pr, prism crystal; se, secretory cavity-like structures; st, staurocytic stoma. Scale bars: 50 µm (B, C, E, F, H, I, K, L); 200 µm (A, D, G, J).

As the three non-flowering specimens, which resemble *Derrissolorioides*, were confirmed to belong to this species based on molecular phylogenetic analyses, they were included as *D.solorioides* in this leaf anatomical study. The results showed that there was little to no variation in leaf anatomical characteristics among specimens from different populations of the same species. The key anatomical traits described in this study were consistent across all examined individuals and populations of both species, indicating stability at the interpopulation level. The leaf anatomical traits of the new *Derris* species and *D.solorioides* are generally consistent with those observed in other *Derris* species (Boonprajan et al. 2024; corresponding author’s observation, unpublished data). However, some distinguishing features were observed in this study. Notably, the new *Derris* species has a thicker leaf texture (subcoriaceous to coriaceous) compared to *D.solorioides* (chartaceous) (Fig. 2F, O). The cross-section at the leaf margin shows a greater degree of curvature in the new species (Fig. 2G) compared to the other (Fig. 2P). The cross-sectional shape of the petiole, rachis, and leaflet midrib also differs between the two species (Fig. 2B, C, E, K, L, N). Both species predominantly exhibit paracytic stomata (Fig. 3C, E, F, K, L) and occasionally anomocytic stomata (Fig. 3L), which is a common trait in *Derris* (Boonprajan et al. 2024; corresponding author’s observation, unpublished data). Interestingly, the staurocytic stomatal type is also occasionally observed but appears to be uniquely present only in the new *Derris* species. Most *Derris* species, including *D.solorioides*, exhibit jigsaw-like or distinctly lobed epidermal cells on both leaf surfaces (Fig. 3H, I, K, L). In contrast, the new species has rectangular or slightly lobed epidermal cells on both leaf surfaces (Fig. 3B, C, E, F). Additionally, unidentified structures resembling secretory cavities (Fig. 3A, D, F) were observed exclusively on the abaxial leaf surface of the new *Derris* species. All leaf anatomical differences between these two species are summarized in Table 2.

**Table 2.** Comparative leaf anatomical characters.

Characters	*Derris* sp. nov.	*Derrissolorioides*
**Transverse section** (Fig. 2)		
Outline of petiole transverse section	horizontal ellipse	vertical ellipse
Cavity in cortex of petiole and petiolule’s pulvinus	indistinct, only small schizogenous cavity present	lysigenous cavity distinctly present
Outline of rachis transverse section	obovate with shallow bi-lobes above	subcircular with shallow bi-lobes above
Outline of midrib transverse section	vertical ellipse	horizontal ellipse
Thickness of midrib cortex	thicker, 155–313 μm	thinner, 87–138 μm
Schizogenous cavity presence in midrib’s cortex	distinctly present	absent
Shape of midrib stele	subcircular	fan-shaped
Mesophyll thickness	thicker, ca. 183 μm	thinner, ca. 135 μm
Palisade mesophyll thickness	1 to 2 cell layers, 50–75 μm	1 to 2 cell layers, 35–60 μm
Spongy mesophyll thickness	105–135 μm	90–110 μm
**Leaf epidermis** (Fig. 3)		
Shape upper/lower epidermal cells	mostly rectangular or shallowly lobed	irregular to jigsaw-like, deeply lobed
Stomata type	typically: paracytic occasionally: anomocytic and staurocytic	typically: paracytic occasionally: anomocytic
Unidentified structures resembling secretory cavities on abaxial leaf surface	present	absent
Druse crystal accumulation	absent or indistinct	present

### ﻿Morphological comparison

As mentioned in the introduction, the new species is partially sympatric with *D.solorioides* and shares some morphological similarities in their vegetative parts, such as bark color and the shape, size, and number of leaflets. However, our detailed morphological observations have revealed several significant differences, particularly in reproductive structures. For instance, *D.solorioides* has a true panicle inflorescence and lacks brachyblasts – the reduced lateral branches of the inflorescence that bear fascicles of florets. In contrast, the new species possesses the longest pseudoraceme/pseudopanicle inflorescence ever recorded in the genus, with each brachyblast bearing up to sixteen florets, the highest number per brachyblast documented in the genus. Additionally, the new species exhibits a denser indumentum on its inflorescence, slightly larger floral parts, and fewer ovules. Moreover, as *Derrisrubrocalyx*, a species from northern Queensland and Papua New Guinea, was recovered as the sister taxon to our putative new species in the Bayesian analysis with relatively high posterior probability (PP = 0.84), we also included it in the morphological comparison. The morphological characters of *D.rubrocalyx* were compiled based on the species description provided in the most recent revision study by Cooper et al. (2024) to highlight key similarities and differences that may support or contrast the molecular findings. A summary of all morphological differences is provided in Table 3.

**Table 3.** Comparative morphological characters.

Characters	*Derrissolorioides*	*Derris* sp. nov.	*Derrisrubrocalyx*
			(According to the revision by Cooper et al. 2024)
Leaflet texture	chartaceous	subcoriaceous to coriaceous	coriaceous
Number of leaflets	5–9	5–7	3–5
Size of terminal leaflet (cm)	6–12.5 by 2.7–5.5	10.3–17.2 by 5.1–10.9	3–17 by 2.5–8.7 (overall leaflet)
Size of lateral leaflet (cm)	4.5–11 by 2.2–5.5	9–14.1 by 4.6–6.9	
Leaflet apex	obtuse or slightly emarginate	emarginate	short or long acuminate (mostly abruptly tapering) and often ultimately minutely retuse
Inflorescence type	true panicle, brachyblast absent	pseudoraceme or pseudopanicle, brachyblast present	pseudoraceme or pseudopanicle, brachyblast present
Inflorescence length (cm)	11–21	44–155	4–36.5
Inflorescence indumentum	glabrous	pubescent	puberulent
Length of inflorescences’ lateral branches (cm)	2.2–15	11–21	up to 14
Brachyblast shape	-	short and swollen at the base (knob-like), to gradually elongate to a cylindrical form	not provided
Brachyblast length (mm)	-	3–12	3–30
Number of flowers per a brachyblast	-	9–16	1–7 (mostly 3- or 4)
Calyx colour	pale green	reddish or with greenish tinge	purple or greenish-red
Calyx indumentum	both sides glabrous, with few hairs near the lobes	outside pubesscent, inside glabrous	outside (abaxially) puberulent and indumentum denser near base and apex, inside (adaxially) puberulent towards apex
Corolla colour	pale green to white	white and gradually turn to pink during maturation	pink
Flower fragrance	fragrant	fragrant	no fragrance detected
Size of standard petals/petal claw length (mm)	5–5.7 by 5.5–7/3.3–3.5	7–10 by 7–7.5/2–2.5	7.5 by 8/1.5
Size of wing petals (mm)	1.7–2.3 by 6–7	5.5–6.3 by ca. 3	7.25 by 2
Wing petals characteristic	slightly curved	straight or slightly curved near apex	strongly revolute
Size of keel petals (mm)	2–2.5 by 5.5–6	5.5–6.5 by 2–3.5	7.25 by 2
Number of ovules per ovary	ca. 8	1–4	2–5
Size of pods (cm)	4.5–8 by 1.8–2.2	5–9.5 by 2–3.5	2.5–11 by 1–2.5
Number of seeds per pod	1–2	1–3	1–4
Seed size (mm)	13–14 by 10–11	9–15 by 6–10	7–10 by 11–14

## ﻿Taxonomic treatment

### ﻿ Derris longiracemosa

Taxon classificationPlantaeFabalesFabaceae

Boonprajan & Sirich. sp. nov.

F60C7428-8D26-5BA6-88CD-A451642C0672

urn:lsid:ipni.org:names:77366492-1

Figs 4 , 5

#### Type.

Thailand • Ratchaburi Province, Photharam District, Tao Pun Sub-district, Wat Khao Chong Phran, ca. 80 m elevation, 13°43'08.8"N, 99°46'21.4"E, 23 December 2021, *Y. Sirichamorn & S. Oncham, YSM2021-36* (holotype BKF!; isotypes K!, L!).

**Figure 4.**
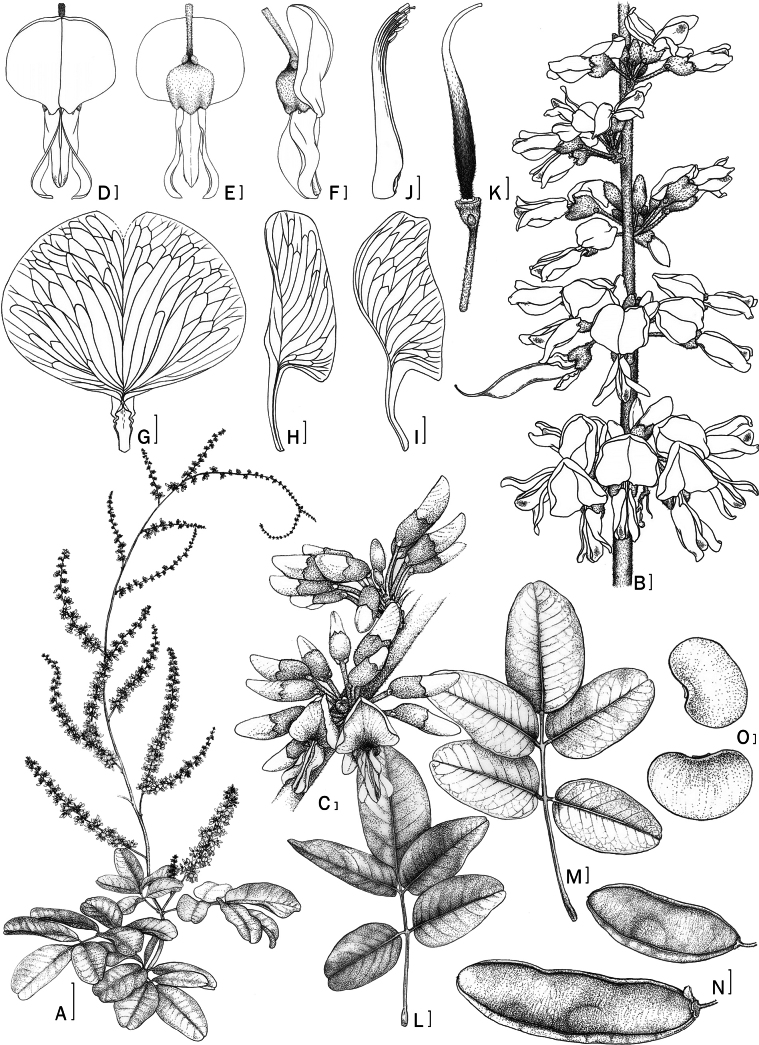
*D.longiracemosa* sp. nov. A. Inflorescence with leaf; B. Close-up of the inflorescence; C. Close-up of the inflorescence (close bud with brachyblast); D–F. Flowers (front and side view); G. Standard petal; H. Keel petal; I. Wing petal; J. Stamens; K. Pistil; L. Adaxial; M. Abaxial leaves; N. Pods; O. Seeds. Scale bars: 5 cm (A); 1 mm (B–K, O); 1 cm (L–N). Drawn by Yotsawate Sirichamorn (A, C, L–O) and Punvarit Boonprajan (B, D–K).

#### Diagnosis.

The species exhibits several morphological traits that distinguish it from the coexisting species *Derrissolorioides*. The texture of the leaflets is subcoriaceous to coriaceous (compared to chartaceous in *D.solorioides*). The leaflet apices are more emarginate. The inflorescences are clearly pseudoracemose or pseudopaniculate with brachyblasts (vs. a true panicle without brachyblasts in *D.solorioides*). These inflorescences can reach up to 155 cm in length, making them the longest recorded in the genus. The peduncle, rachis, and lateral branches of the inflorescence – including brachyblasts, pedicels, and calyces – are pubescent (as opposed to almost glabrous in *D.solorioides*). The number of flowers per brachyblast is also highest, with up to 16 flowers. Petal color changes during maturation, ranging from pure white to pink. The ovary contains fewer ovules, with 1 to 4 ovules (vs. ca. 8 in *D.solorioides*). Additionally, the flowering time is slightly earlier, occurring from November to December (vs. January to February in *D.solorioides*).

**Figure 5.**
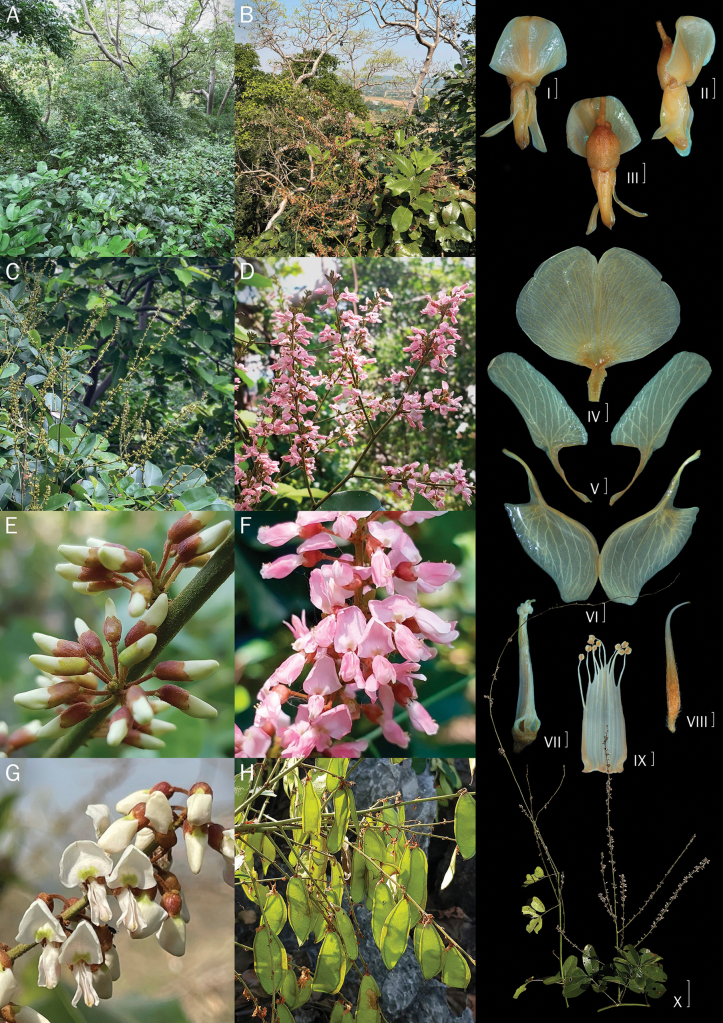
*D.longiracemosa*. A, B. Habit and habitat; C, D. Inflorescence; E. Close-up of inflorescence, showing flowers on brachyblasts; F. Flowers on a late blooming day, turning to a more pink hue; G. Flowers on an early blooming day, displaying white coloration; H. Pods; (I–III) flowers (front and side view); (IV) standard petal; (V) wing petals; (VI) keel petals; (VII) stamens and pistil; (VIII) pistil; (IX) stamens; (X) inflorescence with leaves. Scale bars: 1 mm (IV–IX); 2 mm (I–III); 10 cm (X). Photos by Punvarit Boonprajan (A–C, G, X), Yotsawate Sirichamorn (D–F, H), and Saruta Oncham (I–IX).

#### Description.

Woody climber, bark thin, smooth, pale greyish-brown; twigs glabrous or thinly hairy, lenticellate. ***Stipules*** caducous, triangular, 1.7–2 by 1.6–2 mm, outside glabrescent, margin fimbriate, inside glabrous. ***Leaves*** subcoriaceous to coriaceous; petiole 4.7–13 cm long, grooved above, glabrous or with some scattered hairs, rachis 4–24.5 cm long; pulvinus 6–9 mm long. ***Leaflets*** 5–7; petiolules 4–10 mm long; stipels absent; terminal one elliptic to obovate, 7.5–17.5 by 5–11 cm, base cuneate to round, apex usually emarginate, upper surface and lower surface glabrous, midrib flat or slightly raised above, distinctly raised below, veins flat above, raised below, lateral veins 7–12 pairs, 10–30 mm apart, not reaching the margin but curving towards the apex, sometimes anastomosing near the margin, venation reticulate; lateral ones elliptic, ovate, or obovate, 9–15 by 4.5–7 cm, base broadly cuneate to obtuse, apex shortly emarginate or round, rarely shortly acuminate, upper and lower surface glabrous. ***Inflorescences*** axillary or terminal, pseudoracemes/pseudopanicles, 44–155 cm long, pubescent; peduncle 4–11 cm long, pubescent; lateral branches 23–45 cm long; bracts subtending brachyblasts ovate to triangular, 0.8–1 by 1.2–1.5 mm, outside thinly pubescent and densely pubescent at base, inside glabrous, margin fimbriate. ***Brachyblasts*** knob-like to cylindrical, 3–12 mm long, pubescent, with 9–16 flowers throughout; floral bracts ovate or triangular, ca. 1 by ca. 0.6 mm, outside thinly pubescent and densely pubescent at base, inside glabrous, margin fimbriate; pedicels 4–9 mm long, pubescent; bracteoles at calyx base, ovate or triangular, 0.9 by 0.55–0.65 mm, outside pubescent, inside glabrous. ***Calyx*** reddish or with greenish tinge, cup-shaped, 3.5–4 mm high, outside pubescent, inside glabrous; tube ca. 2 mm long, upper lip with 2 short triangular lobes, 1.5 by 1.5 mm; lateral lobes triangular, 1.5–2 by 1.4–1.8 mm; lower lobe triangular, 1–1.3 by ca. 2 mm. ***Flower*** fragrant. ***Corolla*** white, gradually turning pink during maturation; standard white or pale pinkish with light green central patch, broadly obovate or orbicular, 7–10 by 7–7.5 mm, apex emarginate, basal callosities absent, outside and inside glabrous, claw 2–2.5 mm long; wings white or pale pinkish, oblong, 5.5–6.3 by ca. 3 mm, apex rounded, outside and inside glabrous, upper auricle 0.5–0.75 mm long, lower auricle indistinct, claw 3.2–4 mm long; keel white or pale pinkish, boat-shaped, 5.5–6.5 by 2–3.5 mm, apex rounded, outside glabrous or thinly ciliate at apex, inside glabrous, upper auricle 0.75–1 mm long, lateral pocket 1–2 mm long, claw 2.5–3.5 mm long. ***Stamens*** 8–11 mm long, free part of filaments 3.5–5 mm long, glabrous; anthers 0.3–0.5 by 0.3–0.4 mm, glabrous. ***Disc*** indistinct or annular, up to 0.3 mm long. ***Ovary*** up to 10 mm long, 1–4-ovuled, pubescent; style ca. 4.5 mm long, glabrous but thinly pubescent at base. ***Pods*** elliptic, oblong, or sometimes strap-like, 5–9.5 by 2–3.5 cm, thin, with a wing along both sutures, upper wing 1–5 mm wide, lower wing 0.5–2 mm wide, young pod reddish and gradually turning to light green during maturation, dry pod light brown, glabrescent, seed chamber indistinct but usually slightly darker around the seed. ***Seeds*** 1–3, discoid or bean-shaped, 9–15 by 6–10 by 2–3 mm; hilum central ca. 2 mm long.

#### Phenology.

Flowering from November to December; fruiting from January to February.

#### Etymology.

The specific epithet highlights the species’ distinction of possessing the longest inflorescence ever documented within the genus.

#### Thai name

(assigned here). “Priang prachim” (เปรียงประจิม) consists of two components: *Priang* is an archaic and rarely used noun with an unclear etymology. It has three distinct meanings, one of which refers to a vine or climbing plant; and *Prachim*, a Thai term influenced by the Sanskrit word “*paschimaam*”, meaning “the west.” Thus, Priang Prachim translates to “vines of the west.” The name reflects the plant’s habit as a liana and its occurrence in Thailand’s western forests.

#### Distribution.

**Figure 6.**
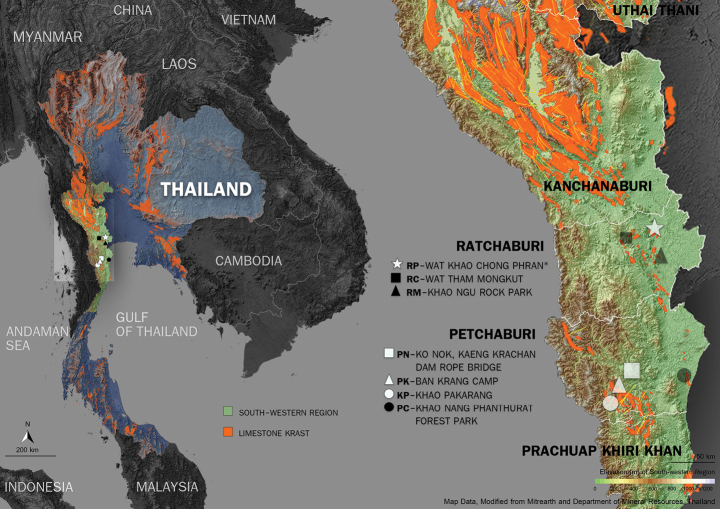
Natural distribution of *Derrislongiracemosa* (white symbols) and *D.solorioides* (black symbols) in southwestern Thailand. The bright orange areas on the map indicate the distribution of limestone karsts in Thailand. The right panel shows a magnified view of the southwestern region, highlighting the localities where the specimens were collected.

Endemic to Southwestern Thailand: Ratchaburi (type, Wat Khao Chong Phran, Photharam District), Phetchaburi (Kaeng Krachan National Park: Kaeng Krachan Dam Rope Bridge, Khao Pakarang, and vicinity of Ban Krang Substation) (Fig. 6).

#### Ecology.

limestone hill (usually found on or near the summit) or occasionally in non-limestone, edge of mixed deciduous forest, 50–450 m.

#### Proposed IUCN conservation assessment.

This new species is known only from a single small limestone hill in Ratchaburi Province and three localities in Kaeng Krachan National Park, Phetchaburi Province. The total estimated number of mature individuals is likely fewer than 2,500, with fewer than 250 mature individuals in each subpopulation. The EOO and AOO are approximately 900 km^2^ and 24 km^2^, respectively. Its type locality, Wat Khao Chong Phran, is also a well-known tourist attraction in Ratchaburi Province, famous for the spectacular evening emergence of over a million bats from a cave. The species is thus threatened by ongoing human disturbance. Based on these factors, we provisionally assess its conservation status as Endangered (EN) under the IUCN Red List criteria B1ab(iii)+B2ab(iii), following the guidelines of the IUCN Standards and Petitions Committee (2022, v. 15.1).

#### Additional specimens examined.

Thailand • Ratchaburi Province: Photharam District, Tao Pun Sub-district, Wat Khao Chong Phran, c. 80 m elevation, 5 February 2022, Y. Sirichamorn & S. Oncham, *YSM2022-1* (BKF, pod specimens).

#### Note.

When this species was first discovered on the limestone hill of Wat Khao Chong Phran, it was initially believed to occur on other nearby limestone formations in Ratchaburi Province. However, despite our extensive field surveys conducted from 2021 until the present, we have not found it on any other limestone hills in this province. Instead, only *Derrissolorioides* has been recorded. Subsequently, the species was rediscovered in two non-limestone habitats within Kaeng Krachan National Park, Phetchaburi Province, raising questions about whether the species is strictly restricted to limestone substrates or may exhibit some degree of ecological plasticity. This uncertainty persisted until it was later found on the summit of Khao Pakarang, a limestone hill in Kaeng Krachan National Park. We hypothesize that the non-limestone populations may have originated from seeds dispersed from limestone-dwelling mother plants. Given that this species produces numerous lightweight pods that are easily carried by the wind, it is possible that some seeds “escaped” and successfully established themselves in non-limestone habitats. Such a pattern raises intriguing questions about the dispersal capacity and ecological flexibility of this taxon, warranting further investigation.

Although *Derrislongiracemosa* and *D.solorioides* are partially sympatric, as they grow in similar limestone environments and exhibit overlapping distributions, notably, they have never been observed coexisting in the same habitat; i.e., in a given locality, only one species is present at a time.

##### ﻿Special note to IUCN status of *Derrissolorioides*

This limestone species of *Derris*, previously classified as Critically Endangered (CR B1a+2a; D) by Sirichamorn et al. (2014), has since been discovered in additional populations across western and central Thailand. As a result, its conservation status warrants reassessment. With the inclusion of these newly documented populations, the extent of occurrence (EOO) is now estimated at 28,200 km^2^, and the area of occupancy (AOO) at 28 km^2^. At least nine localities have been recorded, and the total number of mature individuals is estimated to range between 250 and 1,000. Although the AOO falls within the threshold for Endangered (EN), and the number of locations meets the Vulnerable (VU) threshold under Criterion B, the species does not currently show evidence of continuing decline or extreme fluctuations required to satisfy all subcriteria under Criterion B. Considering the relatively small estimated population size and following the IUCN Red List criteria (IUCN 2024, v. 15.1), we propose reclassifying *D.solorioides* as Vulnerable (VU D1).

### ﻿Addition of *Derrislongiracemosa* to the key to Thai species of *Derris*

The new species is inserted as couplet 14 in an update to the most recently modified key to species of *Derris* in the Flora of Thailand (Sirichamorn 2020; 391–392) and Boonprajan et al. 2024; 72–73).

**Table:** 

1a	Inflorescences paniculate or intermediate forms between panicles and pseudoracemes /pseudopanicles (brachyblasts absent or present but not throughout rachises or lateral branches	**2**
2a	Inflorescences paniculate. Brachyblasts absent. Pedicels glabrous	**3**
3a	Standard 5.5–7 × 5–5.7 mm; pods one-winged	***D.solorioides*** [Note: Compared with *D.longiracemosa*, see couplet 14]
3b	Standard 8–9 × 7–7.5 mm; pods two-winged	***D.marginata***
2b	Inflorescences intermediate between panicles and pseudoracemes/pseudopanicles; brachyblasts present but not throughout rachises or lateral branches; pedicels sericeous or pubescent	**See couplets 4–5 in Sirichamorn (2020: 391–392)**
1b	Inflorescences clearly pseudoracemose or pseudopaniculate (brachyblasts always present throughout rachises or lateral branches)	**6**
6a	Brachyblasts vary in shape and length, usually with more than three flowers throughout. Standard less than 10 mm long, rarely with basal callosities	**7**
7a	Mature leaflets with reddish midribs. Stamen filament sparsely hairy. Anther base with a tuft of hairs	***D.rubricosta***
7b	Mature leaflets without reddish midribs. Stamen filament glabrous. Anther base glabrous	**8**
8a	Pods one-winged or wingless	**9**
9a	Leaflets hirsute to velvety underneath; stipels present	***D.elegans***
9b	Leaflets glabrous underneath; stipels usually absent	**10**
10a	Leaflets 3.3–7.5 by 0.9–3.5 cm; petiolules 3–5 mm long	***D.laotica***
10b	Leaflets 3.5–16 by 1.5–8.5 cm; petiolules 5–10 mm long	***D.trifoliata***
8b	Pods two-winged	**11**
11a	Pods velvety or sericeous	**12**
12a	Leaflets slightly strigose to velvety below, apex rounded, obtuse or cuspidate to short acuminate. Pods with upper wing 4–10 mm wide, lower wing 4–7 mm wide. Northern and North-eastern Thailand	***D.ferruginea***
12b	Leaflets usually strigose to almost glabrous below, apex distinctly acuminate. Pods with upper wing 5–9 mm wide, lower wing 2–4 mm wide. Southern Thailand	***D.pubipetala***
11b	Pods mostly glabrous	**13**
13a	Leaflets 9–11, narrowly obovate, base narrowly cuneate to attenuate	***D.monticola***
13b	Leaflets 3–7 (–9), elliptic ovate or obovate, base cuneate to obtuse	**14**
14a	Inflorescence typically more than 50 cm long (rarely as short as 44 cm in available specimens). Brachyblast with 9–16 flowers, flowers white but gradually turning to pink during maturation	***D.longiracemosa***
14b	Inflorescence typically less than 50 cm long. Flowers 1–8, flowers white, pinkish, or purplish, not distinctly changing color during maturation	**15**
15a	Leaflets sometimes glaucous below. Lateral veins reaching the leaf margin	***D.amoena***
15b	Leaflets never glaucous below. Lateral veins not reaching the leaf margin but curving toward the leaf apex, sometimes forming an intramarginal-like vein	**16**
16a	Leaflets 3–5. Terminal leaflets distinctly longer and wider than lateral ones. Calyx glabrous outside	***D.glabra***
16b	Leaflets 5–7. Terminal leaflets slightly longer but not wider than lateral ones. Calyx thinly sericeous outside	***D.pseudomarginata***
6a	Brachyblasts slender with two or three flowers at the apex. Standard usually more than 10 mm long, usually with basal callosities	**See couplets 17–18 in Sirichamorn (2020: 391–392)**

## Supplementary Material

XML Treatment for Derris longiracemosa

## ﻿Additional information

### Use of AI

No use of AI was reported.

### Author ORCIDs

Punvarit Boonprajan https://orcid.org/0009-0006-7251-1928

Saruta Oncham https://orcid.org/0009-0009-6445-4486

Yotsawate Sirichamorn https://orcid.org/0000-0002-3026-3894

### Data availability

All of the data that support the findings of this study are available in the main text or Supplementary Information.

## Supplementary materials

Supplementary material 1 Voucher specimen and Genbank accession for newly generated sequences reported in the study Data type pdf Explanation note The table includes taxon names, voucher specimen information (collection number, herbarium code, and locality), and corresponding GenBank accession numbers for each DNA region sequenced. These data serve as references for molecular identification and phylogenetic analyses. https://binary.pensoft.net/file/1388325This dataset is made available under the Open Database License (http://opendatacommons.org/licenses/odbl/1.0/). The Open Database License (ODbL) is a license agreement intended to allow users to freely share, modify, and use this Dataset while maintaining this same freedom for others, provided that the original source and author(s) are credited.Punvarit Boonprajan1 Saruta Oncham, Yotsawate Sirichamorn
