# Supplementary material for: ﻿Derrislongiracemosa (Fabaceae), a new species from Thailand with extraordinary limestone adaptations and the longest inflorescences ever recorded
Source: PhytoKeys. 2025 Aug 5;261:13–32. doi: 10.3897/phytokeys.261.156249 (PMC12344443; doi:10.3897/phytokeys.261.156249)
Supplement: Supplementary material 1 — Voucher specimen and Genbank accession for newly generated sequences reported in the study [file phytokeys-261-013_article-156249__-s001.pdf]

Table S1. Voucher specimen and Genbank accession for newly generated sequences reported in the study.

Species	Voucher specimen	Code	Locality	Genbank accession Number		
				trnL-F IGS	*trnK-matK*	ITS/5.8S
Derris sp. (*D. longiracemosa* sp. nov.)	YSM2021-36	RP	Thailand: Wat Khao Chong Phran, Photharam District, Ratchaburi province	PQ586785	PQ586789	PQ61329
	YSM2023-1	KP	Thailand: Khao Pakarang, Kaeng Krachan National Park, Petchaburi province	PQ586786	PQ586790	PQ613293
	YSM2023-2	PK	Thailand: Ban Krang Camp, Kaeng Krachan National Park, Phetchaburi province	PQ586787	PQ586791	PQ613294
	YSM2023-15	PN	Thailand: Ko Nok, Kaeng Krachan Dam Rope Bridge, Krachan National Park, Phetchaburi province	PQ586788	PQ586792	PQ588087
Derris solorioides	YSM2021-33	RC	Thailand: Wat Tham Mongkut, Chom Bueng district, Ratchaburi province	PQ586794	PQ586793	PQ613297
	YSM2021-34	RM	Thailand: Khao Ngu Rock Park, Mueang Ratchaburi district, Ratchaburi province	PQ586795	PQ586796	PQ613298
	YSM2021-35	PC	Thailand: Khao Nang Phanthurat Forest Park, Cha-am district, Phetchaburi province	PQ588141	PQ588142	PQ613296
